# An Integrated *In Vitro–In Silico* Approach for Silver Nanoparticle Dosimetry in Cell Cultures

**DOI:** 10.1007/s10439-020-02449-5

**Published:** 2020-01-13

**Authors:** Daniele Poli, Giorgio Mattei, Nadia Ucciferri, Arti Ahluwalia

**Affiliations:** 1 Research Center E. Piaggio, University of Pisa, Pisa, Italy; 2 Department of Information Engineering, University of Pisa, Pisa, Italy

**Keywords:** Particokinetic model, Diffusion, Dissolution, Sedimentation, Ag nanoparticles

## Abstract

Potential human and environmental hazards resulting from the exposure of living organisms to silver nanoparticles (Ag NPs) have been the subject of intensive discussion in the last decade. Despite the growing use of Ag NPs in biomedical applications, a quantification of the toxic effects as a function of the total silver mass reaching cells (namely, target cell dose) is still needed. To provide a more accurate dose-response analysis, we propose a novel integrated approach combining well-established computational and experimental methodologies. We first used a particokinetic model (ISD3) for providing experimental validation of computed Ag NP sedimentation in static-cuvette experiments. After validation, ISD3 was employed to predict the total mass of silver reaching human endothelial cells and hepatocytes cultured in 96 well plates. Cell viability measured after 24 h of culture was then related to this target cell dose. Our results show that the dose perceived by the cell monolayer after 24 h of exposure is around 85% lower than the administered nominal media concentration. Therefore, accurate dosimetry considering particle characteristics and experimental conditions (e.g., time, size and shape of wells) should be employed for better interpreting effects induced by the amount of silver reaching cells.

**Electronic supplementary material:**

The online version of this article (10.1007/s10439-020-02449-5) contains supplementary material, which is available to authorized users.

## Introduction

Engineered nanomaterials (ENMs) are very successful in the bio-technology industry because of their exceptionally small size and unique physical and chemical properties.^11^,^14^ One of the most widely used ENMs is silver, popularly known for its antimicrobial properties. It can be coated on biomedical devices,^10^ used in medical contexts for personal health care^17^ or biological applications,^36^ and adapted for food products such as kitchen tools, storage containers and cutting boards.^31^ However, despite the growing use of silver nanoparticles (Ag NPs) in the last decade,^1^,^8^,^26^,^44^ potential human and environmental hazards resulting from exposure to Ag NPs continue to be the subject of attention^38^,^54^ owing to their well-documented toxicity both *in vivo* and *in vitro*. In fact, well-established *in vivo*^7^,^13^,^46^ and *in vitro* systems^33^,^45^,^48^ have been proposed for toxicological studies. Mouse and zebrafish models exposed to ENMs, for example, showed nanotoxicity effects on female reproductive and fetal development.^27^,^40^*In vitro*, dose-dependent Ag NPs induced cellular necrosis, inflammation and oxidative stress in living organisms in a size-specific manner.^6^,^16^,^24^,^30^,^34^,^50^ The oxidative stress was further related to the anti-microbial activity of Ag NPs affecting different types of pathogens.^9^,^15^,^39^ Le et al. also discussed short- and long-term toxicity induced by the interaction of Ag NPs with cellular interfaces (e.g., decreased cell viability and apoptosis).^26^ Genetic damage (e.g., DNA breakage) was additionally found within cells interacting with Ag NPs^19^ and associated with the production of reactive oxygen species.^35^,^43^ The interaction of nanoparticles with cellular interfaces can induce other effects such as biophysical changes in the cellular membrane and cytoskeleton, giving rise to changes in cell elasticity, morphology, motility and adhesion.^51^ All these alterations and biophysical changes are often misinterpreted since they are generally reported as a function of the initial silver nanoparticle concentration in liquid media (here referred to as *nominal media concentration*) and not as a function of that actually coming into contact with cells (i.e. *target cell dose*, here defined as the silver concentration reaching cells, including ions and NPs, divided by the total volume of the media). At a given experimental time, this target cell dose is lower than the nominal media concentration because of NP transport (e.g. particle settling in static experiments, Brownian motion) and dissolution in the culture media before reaching cells. Only under ideal conditions (*t* → *∞*) are the two concentrations equal. Therefore, on the basis that the target cell dose is the *cause* of the decrease in cell viability, our aim was to provide a more accurate dose-response analysis by reporting the cellular viability (i.e. *effect*) as a function of the predicted dose coming into contact with cultures.^4^ Specifically, we propose a new approach integrating computational^12^,^28^,^32^ and experimental models.^33^,^45^,^48^ First, we experimentally validated Ag NP sedimentation by comparing the concentrations in static-cuvette experiments with theoretical values computed by an established particokinetic model (ISD3), published by Thomas *et al*.^42^ After verifying that ISD3 correctly predicts the sedimentation process, we proceeded to use this model for estimating the target cell dose (i.e. Ag NPs and dissolved Ag^+^ in cells) perceived by cells in culture. Cell viability was finally related to the computed target cell doses and compared to the nominal media concentrations of Ag NPs initially administered to cells.

## Materials and Methods

### In Vitro–In Silico Pipeline

**Figure 1:**
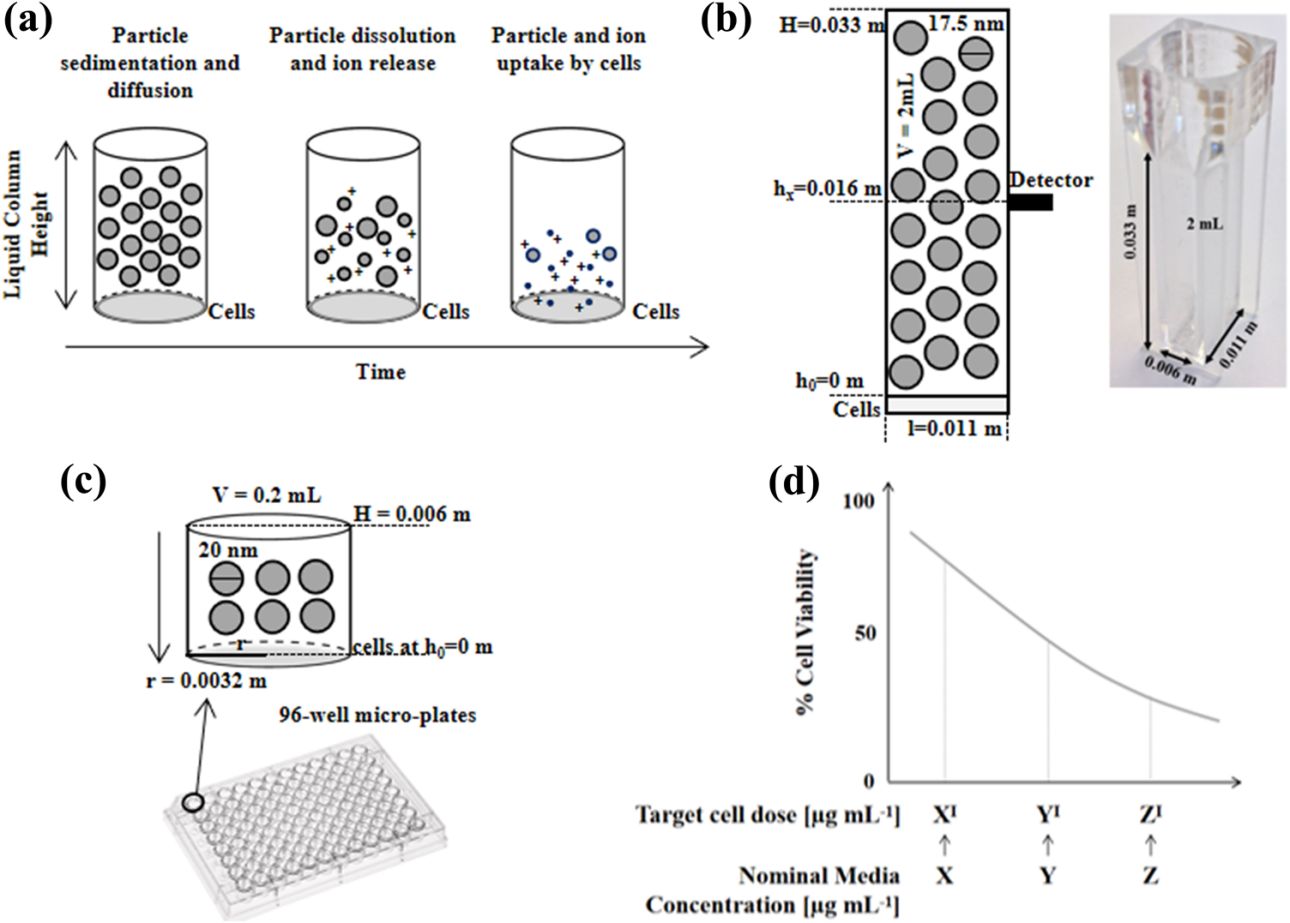
Particokinetics and experimental validation. (**a**) Graphical representation of Ag NP sedimentation, diffusion and dissolution in time. (**b**) Nanoparticle sedimentation in static-cuvette experiments for model validation. (**c**) 96-well micro-plates experiments for evaluating cell toxicity induced by computed effective Ag NP doses. (**d**) Cell viability vs. computed target cell doses at time *t*. Notably, at a finite experimental time *t*, target cell doses (here denoted as *X*′, *Y*′ and *Z*′) are lower than their respective nominal media concentrations (i.e. *X*, *Y* and *Z*).

Figure 1 schematises the integrated *in vitro–in silico* pipeline used in this study. We first adapted ISD3^42^ to our experimental configuration (Fig. 1a).

Then we measured Ag NP sedimentation in static-cuvette experiments and compared the results with those computed by the model (Fig. 1b). In the next step, the model was used to estimate the target cell dose on cell monolayers cultured in 96-microwells for up to 24 h (Fig. 1c). The computed target cell doses as well as the nominal media concentrations of Ag NPs administered to cells were related to cell viability. (Fig. 1d).

### Cell Cultures

Human umbilical vein endothelial cells (HUVECs) and the human hepatoma-derived hepatocyte C3A cell line (ATCC Culture, USA) were used for evaluating nanotoxicity.^45^ Specifically, HUVECs were obtained as described in our previous work^45^ and seeded at a concentration of 20,000 cells cm^−2^ on 1% w/v gelatin coated 96-well plates. Cells were allowed to reach confluence (typically 24 h) before exposure to Ag NPs. C3A hepatocytes were seeded at a density of 200,000 cells cm^−2^ on collagen coated plates and incubated for 24 h before experiments. In view of conducting future co-culture experiments in a flow through system, the same medium was used for Ag NP exposure experiments for both HUVEC and hepatocytes. The cells were cultured in Eagle’s Minimum Essential Medium (EMEM, Lonza Bioscience, Basel, Switzerland), supplemented with 10% Fetal Bovine Serum (FBS, PAA, Pasching, Austria), 1% Penicillin/Streptomycin/Amphotericin B, 2 mM l-Glutamine, 1% non-essential amino acids, 1% MEM vitamins solution (all from Lonza Bioscience, Basel, Switzerland), 10 *µ*g mL^−1^ Endothelial Cell Growth Supplement (ECGS), 10 ng mL^−1^ Human Epidermal Growth Factor (hEGF), 3 ng mL^−1^ basic Fibroblast Growth Factor (bFGF), 1 *µ*g mL^−1^ Hydrocortisone, and 10 *µ*g mL^−1^ Heparin Sodium Salt (all from Sigma-Aldrich, St. Louis, USA).

### Silver Nanoparticles

Ag NM300 from Ras GmbH, an OECD referenced nanomaterial (NM) with a nominal diameter of 20 nm, was purchased as 10% w/w suspension in a aqueous solution containing 7% v/v ammonium nitrate as stabilizing agent and 4% v/v Tween 20 and 4% v/v polyoxyethylene glycerol trioleate, as dispersants. The protocol developed by Klein *et al.*^25^ at the European Commission’s Joint Research Centre (JRC) and employed in several publications was used for a stock solution preparation.^20^,^21^ Ag NPs suspended in the same medium used in our experiments were characterized, applying the methods reported in Refs. 23 and 45, as discussed by JRC.^25^

### Static Sedimentation Experiments

A 2 mL quartz cuvette (Fig. 1b) was filled with the Ag NP suspension and corked to avoid evaporation. Sample absorbance was measured at 414 nm for up to 24 h using a Varian Cary UV spectrophotometer equipped with a 1 mm hole positioned at half height of the cuvette. Nanoparticles were monitored at different nominal media concentrations (i.e. 5, 10, 15, 40, 50, 80 *µ*g mL^−1^). For each Ag NP concentration used, the absorbance at the beginning of the experiment (time 0 h) was used to generate a calibration curve.

### NP Exposure and Toxicity Assay

The medium of both cultures was replaced with 0.2 mL of medium containing homogeneously suspended Ag NPs at different concentrations (from 0 to 100 *µ*g mL^−1^) at experimental time *t* = 0. Cells were then cultured for up to 24 h. To assess cell toxicity, medium containing Ag NPs or dispersant was removed after each incubation time and fresh medium and Alamar reagent (CellTiter-Blue® Promega, Madison, USA) were added. The assay reagent quantitatively measures mitochondrial activity through a cell-permeant fluorescent indicator. Cell viability was obtained from the slope in fluorescence emission within a 2 h time frame measured with a plate-reader (Omega-Fluostar Inc) and expressed as a percentage with respect to cells exposed to 0 *µ*g mL^−1^ Ag NP. For each particle concentration was tested in the absence of cells in order to check for any interaction with the assay.

### The ISD3 Model

ISD3 simulates the sedimentation, diffusion and dissolution of spherical nanoparticles in liquid media.^42^ This particokinetic model was used for predicting silver concentrations (including Ag NPs and Ag^+^ ions) perceived by cultured cells (Fig. 1a). All the equations are reported in the Supplementary materials. In ISD3, the particle number density (*N*) is defined at any time (*t*) and space (*x*) as a function of the NP diameter (*D*_*p*_) and depends on diffusion (coefficient *D*_diff_), sedimentation (characteristic velocity $V_{t}$), and dissolution rate:

1 $$\frac{{\delta N\left( {D_{p} ;x, t} \right)}}{\delta t} = D_{\text{diff}} \left( {D_{p} } \right)\frac{{\delta^{2} N\left( {D_{p} ;x,t} \right)}}{{\delta x^{2} }} - V_{t} \left( {D_{p} } \right)\frac{{\delta N\left( {D_{p} ;x,t} \right)}}{\delta x} - \frac{\delta }{{\delta D_{p} }}\left( {N\left( {D_{p} ;x,t} \right)\frac{{\delta D_{p} }}{\delta t}} \right)$$

**Figure 2:**
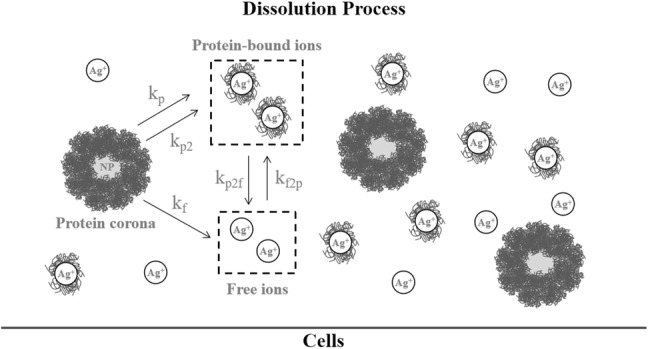
Schematic of Ag NP dissolution process. The dissolution rate constants from Ag NPs to free ions (*k*_f_), protein (*k*_p_) and protein-bound NPs (*k*_p2_) are described, as well as the rate constants for the free ions in solution to protein-bound NPs (*k*_f2p_) and the transfer of ions from protein-bound NPs to free ions (*k*_p2f_).

The dissolution kinetics of Ag NPs are depicted in Fig. 2. Silver NPs are assumed to be immediately surrounded by a protein corona when exposed to cell culture media. Free Ag ions dissolve from the surface of the NPs and may be bound to proteins $\left( {C^{{{\text{diss}},p}} } \right)$ or free $\left( {C^{{{\text{diss}},f}} } \right)$. The ions are taken up by the cells through a membrane diffusion process (eqn. 4 in the Supplementary materials), while Ag NPs are instantaneously taken up by cells. Following the notation used in Ref. 42, $k_{f}$ is the rate constant for the transfer of ions from the particle surface to the free ion state, *k*_*p*_ the rate constant for the slow transfer of ions from the particle surface to the proteins, *k*_*p*2_ the rate constant for the initial fast transfer of silver ions from the particle surface to the protein-bound state, *k*_f2p_ the rate constant for the transfer of free ions from solution to the protein-bound state, and *k*_p2f_ the rate constant for the transfer of ions from the protein-bound state to the free ion state.

A sensitivity analysis conducted by Thomas *et al*. demonstrated that ISD3 can describe the dissolution behavior of silver nanoparticles within a 10% sensitivity range of the kinetic parameters. Because the concentration of serum in the media (10% FBS) and the nominal diameter of Ag NP (20 nm) used in this work are the same as those employed by the authors, all the parameters used here are from Ref. 42 except those in bold in Table 1. The latter were modified for modelling the dissolution process in our static-cuvette and 96-well micro-plates. Should other particles be used, they must first be characterized using the methods listed in Table S1 and S2 in the Supplementary Materials. Further information on the techniques used to obtain the rate constants and the use of the ISD3 Matlab code are also provided therein.

**Table 1:** Particokinetic model inputs.

Parameters	Unit	Cuvette	96-well micro-plate
Liquid media			
Height	m	**0.033**	**0.0066**
Volume	mL	**2**	**0.2**
Temperature	K	310	310
Viscosity	N s m^−2^	0.00074	0.00074
Density	g mL^−1^	1	1
Surface area	m^2^	**6.6 × 10**^**−5**^	**3.3 × 10**^**−5**^
Particle characteristics			
Particle diameter $\left( {D_{p} } \right)$	nm	**17.5**	**17.5**
Primary particle density	g m^−3^	10^7^	10^7^
Effective particle diameter $\left( {D_{p}^{eff} } \right)$	nm	**120**	**120**
Protein corona thickness $\left( {\frac{{D_{p}^{eff} - D_{p} }}{2}} \right)$	nm	**51.25**	**51.25**
Effective density	g m^−3^	1.454×10^6^	1.454×10^6^
Dissolution rate constants			
$k_{f}$	mL m^−2^ h^−1^	6	6
$k_{p}$	mL m^−2^ h^−1^	30	30
$k_{p2}$	mL m^−2^ h^−1^	10^3^	10^3^
$k_{f2p}$	mL g^−2^ h^−1^	1.14 10^4^	1.14 10^4^
$k_{p2f}$	mL g^−2^ h^−1^	1.6 10^4^	1.6 10^4^
Grid spacing, Time and Particle dissolution			
Grid spacing along particle diameter	m	**0.5×10**^**−9**^	**0.5×10**^**−9**^
Number of high grid spacing		**100**	**100**
Time	h	**24**	**24**
Particle dissolution to free ions	mL m^−2^ h^−1^	0.0006×10^−5^	0.0006×10^−5^

Parameters adapted for modelling dissolution in static-cuvette and 96-well micro-plate experiments in bold

### Data Processing

Static-cuvette experiments were performed in duplicate, while 96-well plate *in vitro* experiments were performed in triplicate. Data were processed and the dose-response curves plotted using MATLAB R2019b (Mathworks). Results are expressed as means ± standard deviation.

## Results

The particles have an average size of 17.5 nm with euhedral morphology as measured using transmission electron microscopy (TEM). Finally, the mean effective diameter observed using Nanosight system is 120 nm with monomodal size distribution. Data are summarized in Table 2.

**Table 2:** Ag NP characterization.

Nominal diameter (nm)	TEM		Nanosight analysis	
	Average size (nm)	Morphology	Effective diameter in medium (nm)	Size distribution (PTA analysis)
20	17.5	Euhedral	120	Monomodal

Characterization performed by TEM and single particle tracking analysis (PTA) in the cell culture medium

**Figure 3:**
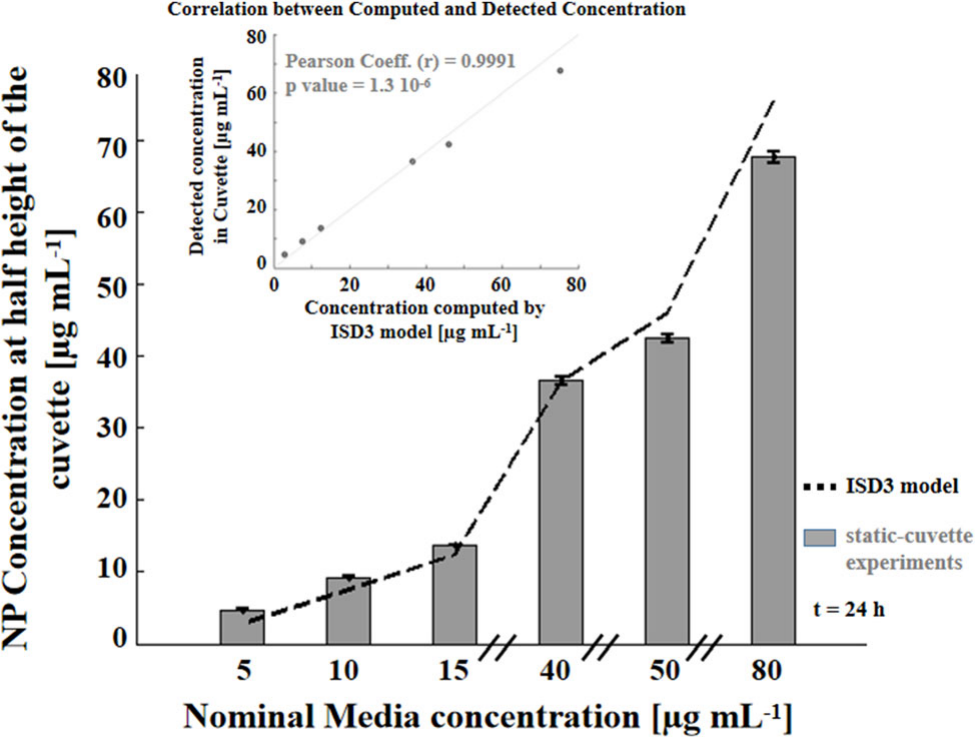
Validation of particle sedimentation in static-cuvette experiments. Ag NP concentrations measured at half height of the cuvette (grey bars) at 24 h fit well with those predicted by ISD3 (black dashed line). The scatter plot (inset) shows a strong correlation (*r* = 0.9991) between the experimental and computed Ag NP concentrations.

We first tracked the Ag NP dissolution and sedimentation process in static-cuvette experiments as a function of the initial administered concentrations (nominal media concentrations). The rationale was to validate ISD3 by quantifying how the predicted nano-particle concentrations fit with those experimentally measured at half height of the cuvette (Fig. 3; grey bars). A high Pearson coefficient (*r* = 0.9991) was observed, indicating a strong correlation between predicted and detected concentrations (inset), confirming the ISD3’s reliability in modelling particle sedimentation.

**Figure 4:**
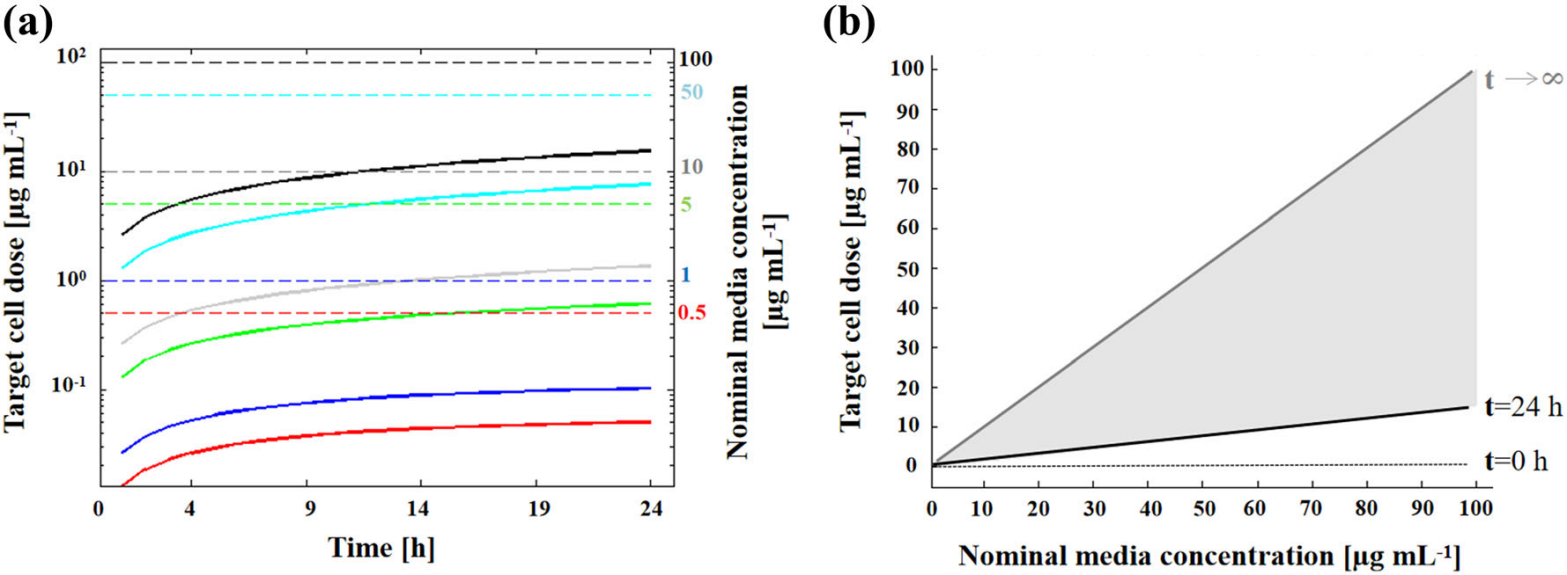
Target cell doses computed over 24 h. (**a**) Target cell doses (solid lines) and nominal media concentrations (dashed lines) as a function of time (red: 0.5 *µ*g mL^−1^; blue 1 *µ*g mL^−1^; green 5 *µ*g mL^−1^; grey 10 *µ*g mL^−1^; cyan 50 *µ*g mL^−1^; black 100 *µ*g mL^−1^). Target cell doses are equal to zero at *t* = 0 (not apparent in the semi-log plot). (**b**) Target cell doses computed at *t* = 24 h (black solid line) vs. nominal media concentrations. Notably, only under ideal conditions (*t* → *∞*) are these two concentrations equal (grey solid line). The black dotted line denotes target cell doses at t = 0 h, which are all null regardless of the nominal media concentration.

Subsequently, we compared predicted target doses reaching the cells (Fig. 4a, solid lines) with different Ag NP nominal media concentrations (dashed lines) over 24 h. The rationale was to provide a more accurate dose-response analysis since the amount of silver ions and Ag NPs coming into contact with cells at a given time is lower than the nominal media concentration administered because particles dissolve and settle. In fact, as represented by the vertical distance between black and grey lines in Fig. 4b, the target cell doses were much lower than their respective nominal media concentrations at the end of our experiment (24 h). These differences, here expressed as Δ = (nominal media concentration − target cell dose)/(nominal media concentration), are reported in Table 3. As expected, the target cell doses were proportional to their respective nominal media concentrations.

**Table 3:** Nominal Ag media concentrations vs. Target cell doses.

Nominal media Ag concentration (*µ*g mL^−1^)	Ag^+^ ions in cells (*µ*g mL^−1^)	Ag particles in cells (*µ*g mL^−1^)	Total target cell Ag dose (*µ*g mL^−1^)	Δ (%)
0.5	0.003	0.047	0.050	89.99
1	0.006	0.096	0.103	89.73
5	0.018	0.594	0.612	87.75
10	0.02	1.341	1.361	86.38
50	0.032	7.612	7.644	84.71
100	0.042	15.495	15.537	84.46

Target cell doses (i.e. Ag NPs and dissolved Ag^+^ in cells) are about 85% lower than their respective nominal media concentrations (i.e. the initial administered doses) at 24 h

**Figure 5:**
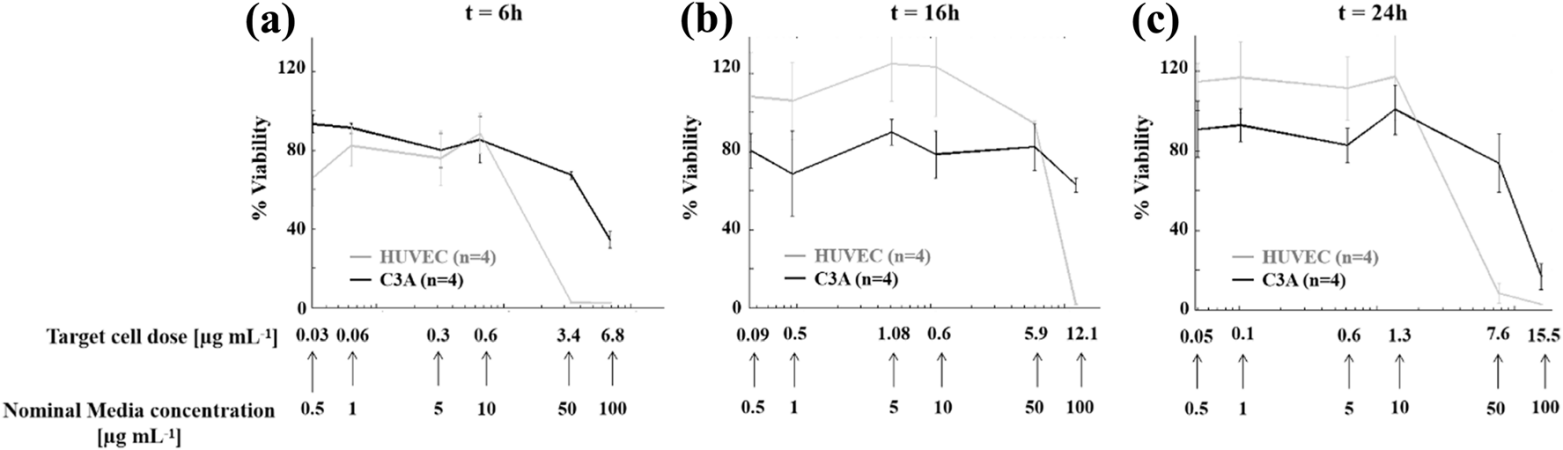
HUVEC (gray line) and C3A (black line) viability as a function of increasing target cell doses. Panels show the viability as measured by the Alamar blue assay at 6 h (**a**), 16 h (**b**) and 24 h (**c**). Data shown as mean ± std (*n* = 4).

Having predicted target cell doses during the entire exposure period (i.e., from 0 to 24 h) and quantified differences with increased nominal media concentrations (Δ values), we investigated time-dependent nanotoxicity effects induced by the effective silver concentrations coming into contact with cells reporting HUVEC and C3A viability at 6 h (Fig. 5a), 16 h (Fig. 5b) and 24 h (Fig. 5c) of culture as a function of the target cell doses. Both HUVEC (gray line) and C3A (black line) viability decreased with increasing target (and nominal) cell dose. From the graphs it is also evident that the Δ has implications on the estimation of the IC50 values. In fact, referring to the nominal media concentration, the IC50 value at 24 h is estimated as 41.04 *µ*g mL^−1^ for HUVEC and 54.78 *µ*g mL^−1^ for C3A. However, if one considers the target cell dose (actually experienced by cells), the IC50 drops significantly (~ 80%): 8.13 *µ*g mL^−1^ for HUVEC and 10.95 *µ*g mL^−1^ for C3A.

## Discussion

An integrated pipeline based on computational (ISD3) and experimental approaches to provide more accurate dose-response analyses and to minimize time-consuming, expensive and ethically sensitive *in vivo* tests^52^ is described. Having verified the accuracy of ISD3 through sedimentation experiments, we evaluated the viability of HUVEC and C3A cells as a function of the total silver mass reaching cells (namely, target cell dose). The results show that target cell doses computed at 24 h are much lower (85%) than the nominal media concentrations initially administered to the cells (*t* = 0 h), thus the nanotoxic effects induced by Ag NP exposure may be underestimated if they are related to the initial administered concentrations.

Much uncertainty still remains regarding which constituent (i.e., NPs, free and protein-bound ions) contributes to cellular toxicity.^2^,^37^ Bouwmeester *et al*.^5^ as well as Wang *et al*.^49^ suggest that exposure to silver ions formed extracellularly is responsible for observed toxic effects. Conversely, other groups assume that the internalized nanoparticles (i.e. NPs taken up by cells) undergo rapid dissolution resulting in silver ions inducing toxicity.^18^,^41^ Therefore, to take into account both NP and ion related toxic effects, the target cell dose in this work includes both dissolved ions and particles reaching cells (i.e. Ag NPs + dissolved Ag^+^ in cells).

Some other fundamental concerns in *in vitro* nanotoxicology still need to be addressed before data from cell culture experiments can be considered reliable for predicting NP toxicity. The ISD3 model, for example, assumes Ag NPs are immediately absorbed once the cell surface is reached. Furthermore, ISD3 does not simulate the intercellular dissolution of NPs once they have been taken up by the cells and it is limited to a 2 dimensional cell configuration, which is not considered “physiologically relevant”. By modelling the kinetics involved within the three-dimensional geometries of cellular constructs, we should be able to predict the Ag NP uptake through multiple layers in a more realistic *in vivo*-like multilayer configuration. Moreover, the computed target cell doses sequentially absorbed “layer by layer” would allow an even more accurate analysis of the nanotoxicity effects. In this regard, the three-dimensional and scaffold-less cellular aggregates in the form of “organoids”, “microtissues” or “spheroids” which resemble the cytoarchitectural arrangement of human tissues (e.g., liver, lung and specific brain regions) are currently being used as more physiologically relevant *in vitro* systems for experimental validation.^29^ For example, Kermanizadeh *et al*.^22^ have investigated three-dimensional human liver microtissue models, exposing them to different types of nanomaterials (including Ag NPs). They used Ag NP concentrations within the range employed here, demonstrating that repeated exposure is more damaging to the liver tissue in comparison to a single exposure. In this context, as there is some interest in the effects of chronic exposure, it would be useful to incorporate dosing regimens into ISD3. The boundary conditions, as well as all variables regarding nanoparticles and dissolved ions in liquid medium, could be updated taking into account multiple doses which are sequentially exposed to the cells and change over every grid point in space at any simulation time. Under the assumption that the chemical equilibrium in the system depends principally on the concentration of dissolved silver ions in liquid $\left( {C^{{{\text{diss}},f}} } \right)$ and in cells $\left( {C^{{{\text{diss}},c}} } \right)$,^3^ we could iteratively add $C^{{{\text{diss}},f}}$ and subtract $C^{{{\text{diss}},c}}$ at any given time in space (Eqs. 3 and 4, respectively, in the Supplementary Materials) and then recalculate the cumulative particle number density. The application of the model to chronic exposure regimens is currently being validated using the same framework as described herein.

It should be noted that for the model configuration described in our work, we would need to compute and experimentally validate not only the NPs diffusing towards the cells cultured on the bottom of the wells, but also those particles coming into contact with the cells adjacent to the walls. To the best of our knowledge, this second computational aspect has been exclusively provided by Bohemert *et al*.^4^ They indeed modelled the dosimetry and the exposure to nanoparticles of Caco-2 cells forming a confluent monolayer on the bottom of the cell culture dish, but also growing up its encircling walls.

Another aspect to consider is that Ag NP sedimentation and cell toxicity were modelled and experimentally evaluated under static conditions. It would be interesting to extend the combined computational–experimental approach to dynamic experiments, implemented with a modular bioreactor similar to that described in our previous work^47^ or a microfluidic system.^53^ A multiphysics computational approach could be used to model the combined effects of particle sedimentation, diffusion, dissolution and convection as a function of the involved fluxes characterizing the dynamics of this type of more “physiologically relevant” *in vitro* model. It would also be interesting to quantify the effects of absorbed nanoparticles of different materials and diameter sizes. These features, including the protein corona on the surface of the nanoparticles (here represented by a 51.25 nm thick protein layer; see Table 1), are known to modulate nanotoxicity.^4^,^42^

In conclusion, combining computational and experimental approaches, we provide more reliable dose–response analyses and hazard assessment. This study paves the way for the development of a methodological framework which combines the expertise of modellers, toxicologists and scientists studying nanotechnology and nanoscience to enable good experimental design and more accurate *in vitro* to *in vivo* data extrapolation.

## Electronic supplementary material

Below is the link to the electronic supplementary material.

Supplementary material (PDF 258 kb)
