# Correction to: An Integrated *In Vitro*–*In Silico* Approach for Silver Nanoparticle Dosimetry in Cell Cultures

**DOI:** 10.1007/s10439-020-02463-7

**Published:** 2020-01-30

**Authors:** Daniele Poli, Giorgio Mattei, Nadia Ucciferri, Arti Ahluwalia

**Affiliations:** 1 Research Center E. Piaggio, University of Pisa, Pisa, Italy; 2 Department of Information Engineering, University of Pisa, Pisa, Italy

## Correction to: Annals of Biomedical Engineering (2020) 10.1007/s10439-020-02449-5

The article An Integrated *In Vitro*–*In Silico* Approach for Silver Nanoparticle Dosimetry in Cell Cultures, written by Ahluwalia *et al*., was originally published electronically on the publisher’s internet portal (currently SpringerLink) on 13 January 2020 without open access. With the author(s)’ decision to opt for Open Choice the copyright of the article changed on 29 January to © The Author(s) 2020 and the article is forthwith distributed under a Creative Commons Attribution 4.0 International License (https://creativecommons.org/licenses/by/4.0/), which permits use, sharing, adaptation, distribution and reproduction in any medium or format, as long as you give appropriate credit to the original author(s) and the source, provide a link to the Creative Commons licence, and indicate if changes were made.

