# Supplementary material for: An Integrated In Vitro–In Silico Approach for Silver Nanoparticle Dosimetry in Cell Cultures
Source: Ann Biomed Eng. 2020 Jan 13;48(4):1271–80. doi: 10.1007/s10439-020-02449-5 (PMC7089903; doi:10.1007/s10439-020-02449-5)
Supplement: Supplementary file 1 — Supplementary material (PDF 258 kb) [file 10439_2020_2449_MOESM1_ESM.pdf]

# Supplementary Materials

# An integrated *in vitro – in silico* approach for silver nanoparticle dosimetry in cell cultures

Daniele Poli[1], Giorgio Mattei[2], Nadia Ucciferri[1] and Arti Ahluwalia[1, 2]

[1]Research Center E. Piaggio, University of Pisa, Pisa, Italy; [2]Department of Information Engineering, University of Pisa, Pisa, Italy

## Ag NP characterization

Particles were dispersed in the complete cell culture medium. The same dispersion protocol was followed for all exposure experiments and when characterizing possible agglomeration tendencies and stability of dispersions. Ag NPs were stored as viscous liquid at the concentration of 10% (w/w) dispersed in water (75%) with a stabilizing agent (ammonium nitrate 7%) and emulsifiers (Tween 20 and polyoxyethylene glycerol trioleate, 4% each). For preparation of a stock solution we used the protocol provided by the ENPRA project (www.enpra.eu): ~100 mg of silver particle solution was weighed and dispersed in ~38 mL of water supplemented with 2% FBS to a final concentration of 2.56 mg $mL^{-1}$.  Immediately after preparation the stock solution was sonicated for 16 min in an ultrasonic bath (Bandelin electronic, Berlin, Germany) at 200 W. Dilutions between 0 and 80 $\mu$g $mL^{-1}$ were prepared in the complete cell culture medium. Before use samples were again sonicated for 15 min.

Particle hydrodynamic diameter was determined via dynamic light scattering using Malvern Zeta sizer nano (Malvern Instruments, Herrenberg, Germany). Size and polydispersity were determined at time points 0, 1, 2, 4, 6, 8 and 24 hours after preparation of the respective dilution. Between measurements, dilutions were kept at room temperature and protected from light. Diluted samples were again sonicated for 16 minutes in an ultrasonic bath. For each time point, two independent samples were measured three times each and mean and standard deviation are reported.

Size distribution and stability were also investigated using NanoSight LM10 instrument (NanoSight Limited, Amesbury, UK) as described in Ucciferri et al (2014). The system is based on dynamic light scattering but uses single particle tracking analysis. At each time point, three measurements were performed; mean value and standard deviation were calculated. Here, sample data at 0, 8 and 24 h are reported (Figure S1).

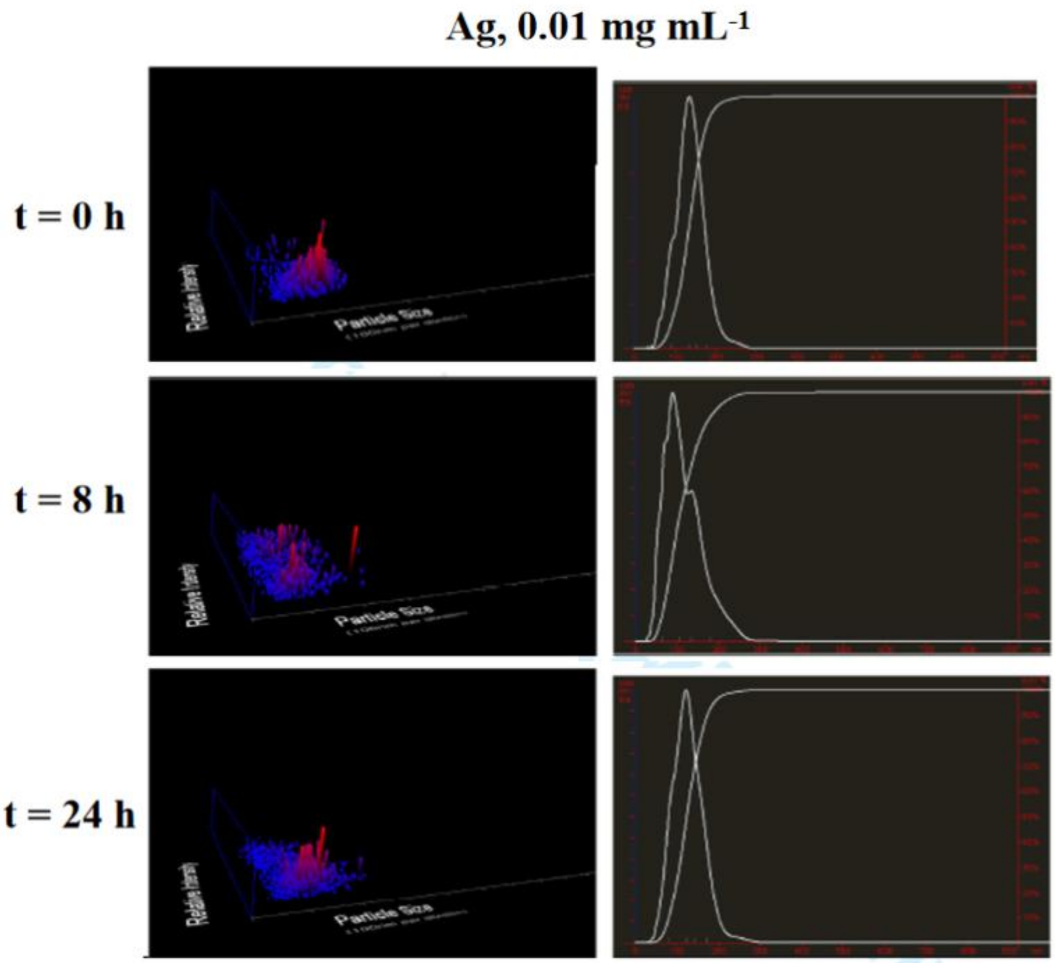


**Figure S1.** Ag NPS tracking at 0 h, 8 h and 24 h.

**ISD3 model**

The ISD3 model developed by Thomas et al. (2018) defines the particle number density ($N$) at any time $t$ and space $x$ as a function of the NP diameter $D_p$ and depends on diffusion (coefficient $D_{diff}$), sedimentation (characteristic velocity $V_t$), and dissolution rate (see eqn. 1 in the manuscript).

The Stoke-Einstein equation is used for quantifying $D_{diff}$ as follows:

$$D_{diff} = \frac{RT}{3N_A \pi \mu D_p} \quad (1)$$

where $R$ is Boltzmann's constant, $T$ the medium temperature, $N_A$ the Avogadro's number and $\mu$ the media viscosity.

$V_t$ is based on Stoke's equation as follows:

$$V_t = \frac{g(\rho_p - \rho_f)D_p^2}{18\mu} \quad (2)$$

where $g$ is the acceleration due to gravity, $\rho_p$ the particle density and $\rho_f$ the medium density.

Cellular uptake of both Ag NPs and $Ag^+$ ions is also modeled. In particular, ISD3 assumes that NPs are instantaneously taken up by cells when they reach the membrane, while cell uptake of ions is explicitly modeled as a membrane diffusion process. The concentrations of free ions ($C^{diss,f}$) and ions in cells ($C^{diss,c}$) are defined as follows:

$$\frac{dC^{diss,f}}{dt} = \frac{k_f A(t)}{V}\left(C_{sat}^{diss,f} - C^{diss,f}\right) \quad (3)$$

$$\frac{dC^{diss,c}}{dt} = \frac{D_{diff} S}{V}\left(\frac{C^{diss,f} + C^{diss,p} - \frac{C^{diss,c}\frac{V}{V_c}}{P_c}}{D_r}\right) \quad (4)$$

where $k_f$ is the rate constant for the transfer of ions from the particle surface to the free ion state, $A$ the surface particle area, $V$ the media volume, $C_{sat}^{dis,f}$ the saturated ion concentration, $S$ the total cell surface area, $C^{diss,p}$ the protein-bound ion concentration, $V_c$ the cell volume, $P_c$ the partition coefficient, and $D_r$ the cell membrane thickness.

Tables S1 and S2 list the ISD3 parameters and summarize how they can be obtained. In particular, liquid media and grid specification, as well as time integration and cellular characteristics, are directly extracted from the experimental conditions. As mentioned in the manuscript, $k_p$ is the rate constant for the slow transfer of ions from the particle surface to the proteins, $k_{p2}$ the rate constant for the initial fast transfer of silver ions from the particle surface to the protein-bound state, $k_{f2p}$ the rate constant for the transfer of free ions from solution to the protein-bound state, and $k_{p2f}$ the rate constant for the ion transfer from the protein-bound state to the free ion state. The MATLAB code of ISD3 model is available from Thomas et al. (2018) and can be downloaded at https://nanodose.pnnl.gov. The boundary conditions of the experimental system can be easily updated by modifying the input data defined and discussed within the MATLAB script named "inputdata.m". Right click and run the "isd3.m" file for beginning the simulations. Outputs at any time in space, such as particles and/or ions in liquid and/or on cells, are finally saved into a MATLAB and EXCEL file.

| | Parameter | Unit | Code Line | Methods | Ref. |
|---|---|---|---|---|---|
| Liquid Media | Dish depth | m | 51 | Directly extracted from the experimental conditions | 47 |
| | Volume | mL | 52 | | |
| | Temperature | K | 53 | | |
| | Viscosity | N s $m^{-2}$ | 54 | | |
| | Density | g $mL^{-1}$ | 55 | | |
| Primary Particle | Hydrodynamic diameter Hydrodynamic diameter | nm | 64 | Measured using Dynamic Light Scattering (DLS) in the medium of interest. It is calculated from the intensity weighted average translational diffusion coefficient by using cumulant analysis on the autocorrelation function using vendor provided software. An additional method can be Fluctuation correlation spectroscopy (FCS) . | 15, 47 |
| Protein-coated Particle | Effective density | g $mL^{-1}$ | 71 | Measured via the volumetric centrifugation method (VCM). A sample of nanoparticle suspension can be also centrifuged in a packet cell volume (PCV) tube to produce a pellet, the volume of which can be measured and used to estimate the effective density of the nanoparticles in suspension | 12, 47 |
| | Protein corona | nm | 72 | The thickness of the protein layers (or protein corona) is assumed to be to ½ the difference in diameters measured by DLS and Transmission Electron Microscopy (TEM), producing an effective particle size equal to the value measured by DLS. Additional methods are Differential centrifugal sedimentation (DCS); Size exclusion chromatography (SEC); Isothermal calorimetry (ITC); Mass spectroscopy; Circular dichroism (CD); Fluorescence quenching; Surface plasmon resonance (SPR). | 7,13, 16,29 ,33,5 3 47 |
| | Effective diameter | nm | 78 | The effective diameter is measured by combining the hydrodynamic diameter and the protein corona as follows: Effective diameter = Hydrodynamic diameter + (2 * protein corona) . | |
| Grid, Boundary conditions | Grid spacing along diameter axis | m | 120 | According to the experimental conditions and the researchers requests. | 12, 47 |
| Time | Number of grid points in x-axis | # | 126 | According to the researchers requests. | |
| | Simulation Time | h | 120 | | |
| | Number of time steps | # | 121 | | |

**Table S1. ISD3 parameters.** 1) Liquid media characteristics; 2) Primary and protein-coated particle characteristics; 3) Grid specification and boundary conditions; 5) Time integration inputs. Lines of the original code defining these parameters are specified to facilitate data entry for first time users of the software.

<table>
<tr><th></th><th>Parameter</th><th>Unit</th><th>Code Line</th><th>Methods</th><th>Ref.</th></tr>
<tr><td rowspan="5">Dissolution</td><td>$k_f$</td><td>ml $m^{-2}$ $h^{-1}$</td><td>142</td><td rowspan="5">These rate constants, as well as the saturated concentration of free ions in solution ($C_{sat}$), the concentration of binding sites available on the proteins per FBS for the slow binding period of the ions ($n$) and the concentration of binding sites available on the proteins per FBS for the fast binding period of the ions ($n_2$), were estimated by fitting experimental data measured in cell culture media with the model. Ion release can be also measured by Atomic Absorption Spectroscopy (AAS).</td><td rowspan="5">4, 12, 32, 47</td></tr>
<tr><td>$k_p$</td><td>ml $m^{-2}$ $h^{-1}$</td><td>147</td></tr>
<tr><td>$k_{p2}$</td><td>ml $m^{-2}$ $h^{-1}$</td><td>150</td></tr>
<tr><td>$k_{p2f}$</td><td>ml $g^{-2}$ $h^{-1}$</td><td>155</td></tr>
<tr><td>$k_{f2p}$</td><td>ml $g^{-2}$ $h^{-1}$</td><td>153</td></tr>
<tr><td rowspan="4">Cellular uptake of ions</td><td>Ion partition coefficient</td><td></td><td>171</td><td rowspan="4">The media silver ion partition coefficient and the diffusion coefficient were estimated by fitting the silver levels measured in cells by means of ICP-MS with the model. The thickness of cell membrane and the cell volume used by Thomas and co-workers were specific for RAW 264.7 macrophage cells.</td><td rowspan="4">47</td></tr>
<tr><td>Diffusion coefficient</td><td>$m^2$ $h^{-1}$</td><td>170</td></tr>
<tr><td>thickness of cell membrane</td><td>nm</td><td>169</td></tr>
<tr><td>Cell volume</td><td>mL</td><td>172</td></tr>
</table>

**Table S2.** Dissolution model inputs and parameters for cellular uptake of ions. As table S1, lines of the original code are specified for data entry.
